# A Recipe for Disaster – Sodium Bicarbonate Overdose

**DOI:** 10.21980/J8MW85

**Published:** 2025-07-31

**Authors:** Adeola A Kosoko, Amara Ogoke, Kyle Vogt

**Affiliations:** * University of Texas Health Science Center at Houston, McGovern School of Medicine, Department of Emergency Medicine, Houston, TX; ^ Emergency Medicine Physician, Houston, TX

## Abstract

**Audience:**

Emergency medicine residents post-graduate years 1–4

**Introduction:**

Sodium bicarbonate is a compound found commonplace in many households and in many products. It lends itself to countless everyday uses including cooking and cleaning. Physicians prescribe sodium bicarbonate regularly both on- and off-label regularly for various ailments.^1^ Due to the ubiquitous way both emergency physicians and the general public use and are exposed to sodium bicarbonate, we wanted to prepare learners to identify and appropriately manage sodium bicarbonate toxicity.

Obtaining a thorough history not just of medications prescribed but of significant ingestions or infusions is critical in diagnosing this toxicity. Furthermore, acute sodium bicarbonate overdose should be considered in a patient who may present with severe metabolic alkalosis.^1^ This case outlines the common signs and symptoms of a patient with acute sodium bicarbonate toxicity and reviews the management of sodium bicarbonate toxicity.

**Educational Objectives:**

At the end of this oral board session, learners will be able to: 1) obtain a history which includes medications and other supplements used by the patient, 2) interpret a prolonged QTc, 3) diagnose metabolic alkalosis due to sodium bicarbonate toxicity, and 4) manage sodium bicarbonate toxicity with fluid and electrolyte resuscitation.

**Educational Methods:**

This case was presented to learners using the typical format for the American Board of Emergency Medicine (ABEM) oral certification examination, a standardized test of emergency medicine knowledge. This educational format allows exploration of the evaluation, workup, and management of the rare case of a patient with sodium bicarbonate toxicity in a safe learning environment.

For faculty, this case can act as an assessment of an emergency medicine resident’s critical thinking skills as they progress through residency. Oral board testing is a key part of resident learning because it prompts residents to apply their learning in a low-stakes environment, both in preparation for the oral certification examination and for clinical practice.

**Research Methods:**

Immediately after the learners completed the oral boards case and debriefing, feedback was solicited using Google forms, a free and open-access online tool. The participants’ feedback was recorded regarding the educational value of the case, using a Likert scale (1–5), and the form also requested feedback about the case, including what was beneficial, and suggestions for improvement.

**Results:**

Twenty-six residents in total participated in this oral boards case and five faculty participated as facilitators. All participating faculty gave verbal feedback. After participating in the case, thirteen residents who completed the feedback form described a score of 4 and 5 on the Likert scale (1–5, 1 = very unfamiliar, 5 = very familiar) regarding diagnosing and managing sodium bicarbonate toxicity.

**Discussion:**

Sodium bicarbonate toxicity is a true medical emergency at the intersection of multiple bodily systems but particularly that of managing fluids and electrolytes. It requires timely diagnosis and management, albeit the occurrence of the toxicity is rare. Acidosis is a far more common occurrence in emergency medicine. This case allows resident learners to explore a rare acid/base imbalance. The educational content of this oral boards case was effective based on the reports of the learners’ familiarity with the subject before and after working through the case. In addition, many residents reported that this educational technique was a good way to learn about a rare patient presentation, diagnosis, and management. We learned that this case was considered more difficult compared to cases focusing on emergency core content diagnoses. However, though it is difficult, the learners appreciated putting together skills they’ve learned from core content and basic emergency medicine patient care concepts to work through this case, which they considered applicable to their future patient care.

**Topics:**

Sodium bicarbonate, baking soda, alkalosis, hypokalemia, hypernatremia, toxicology, prolonged QTc, oral board case.

## USER GUIDE

**Table:** 

List of Resources:	
Abstract	1
User Guide	3
For Examiner Only	7
Oral Boards Assessment	14
Stimulus	17
Debriefing and Evaluation Pearls	29

**Learner Audience:**

Interns, Junior Residents, Senior Residents

**Time Required for Implementation:**

Case: 15 minutes

Debriefing: 5 – 10 minutes

**Recommended number of learners per instructor:**

One “tester” at a time who will navigate the case with the instructor as the spokesperson. If other learners are present, they can observe the tester.

**Topics:**

Sodium bicarbonate, baking soda, metabolic alkalosis, hypokalemia, hypernatremia, toxicology, prolonged QTc, oral board case

**Objectives:**

By the end of this oral boards case, the learner will be able to:

1. Obtain a history which includes medications and other supplements used by the patient
2. Interpret a prolonged QTc
3. Diagnose metabolic alkalosis due to sodium bicarbonate toxicity
4. Manage sodium bicarbonate toxicity with fluid and electrolyte resuscitation

### Linked objectives, methods and results

This oral boards case provides an opportunity to review the diagnosis and management of a patient experiencing sodium bicarbonate toxicity in a safe learning environment simulating many aspects of the typical emergency clinical environment in the ABEM oral board testing format. We chose an oral board case as our educational method to allow the learners to obtain information rapidly and have ample time for debriefing and discussion of the case. Though with some minor adjustments, the case can be modified to a simulation case, we anticipate significantly more opportunities for distractions in workup and management in simulation which are less prevalent in an oral boards case (eg, airway intubation, confusing confederates, lumbar puncture).

The case has the learner simulate management of a critically ill patient by obtaining a thorough and timely history of medications and other supplements used (Objective 1). The learners should correctly interpret a prolonged QTc from an electrocardiogram (ECG) presented (Objective 2). The learners should pursue a broad medical evaluation of the patient presenting with undifferentiated altered mental status. As various data points are presented by the educational instructor, the blood gas and metabolic profile with the abnormal ECG should facilitate a diagnosis of metabolic alkalosis due to sodium bicarbonate toxicity (Objective 3). With the appropriate diagnosis, the learners should request appropriate medical intervention of intravenous fluids and electrolyte replacement to manage sodium bicarbonate toxicity (Objective 4).

### Results and tips for successful implementation

This oral boards case was presented to 26 residents within the same residency program. It was presented in small groups of up to five residents, each with one resident navigating the case with a faculty member, while their colleagues observed their oral board test-taking technique and medical decision-making. The entire group participated in the debriefing, which focused on notifying the resident of the critical actions of the case, discussing test-taking techniques, and reviewing the diagnosis and management of sodium bicarbonate toxicity. The resident navigating the case with the faculty member was scored against the critical actions and the milestone assessments for their own edification. The group setting helped to alleviate the unfamiliarity with the topic of sodium bicarbonate toxicity of most participants, in that they were allowed to ask for help from colleagues when they felt puzzled, or they could continue as though it were a typical test if they were more comfortable. More active learning occurred with the test taker and more passive learning with the observers. Nonetheless, all residents were invited to give feedback on the case regardless of their role. Half of the residents gave feedback by an anonymous online Google form, which only elicited their year in training as a demographic descriptor.

**Figure 1:**
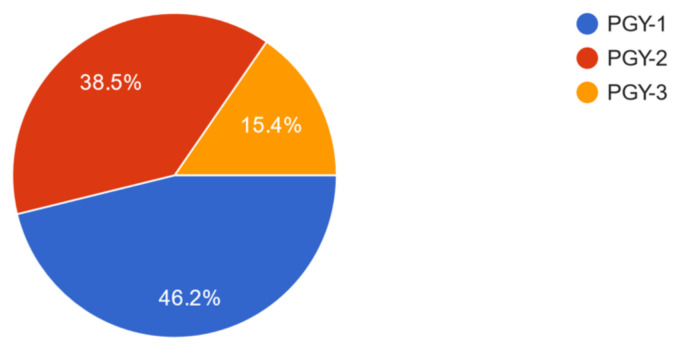
Learners participating in oral boards case by year of training. Post graduate year, PGY.

Thirteen residents (50% of participants) completed the feedback form and provided their year in training information (Figure 1).

**Figure 2:**
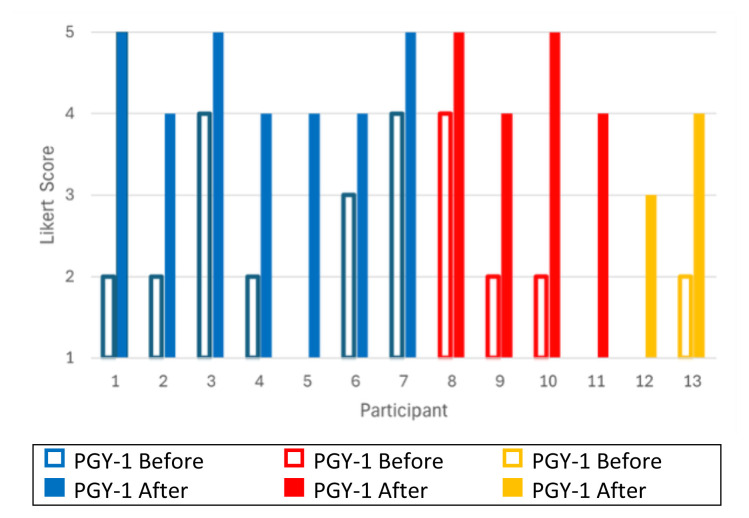
Learners describe familiarity with **DIAGNOSIS** of bicarbonate toxicity before and after this oral boards case by Likert scale (1–5, 1 = very unfamiliar, 5 = very familiar). Post graduate year, PGY.

Score value of residents’ familiarity with diagnosis of sodium bicarbonate toxicity was 2 on a Likert scale (1–5, 1 = very unfamiliar, 5 = very familiar) before the oral boards case and 4 and 5 after the oral boards case (Figure 2).

**Figure 3:**
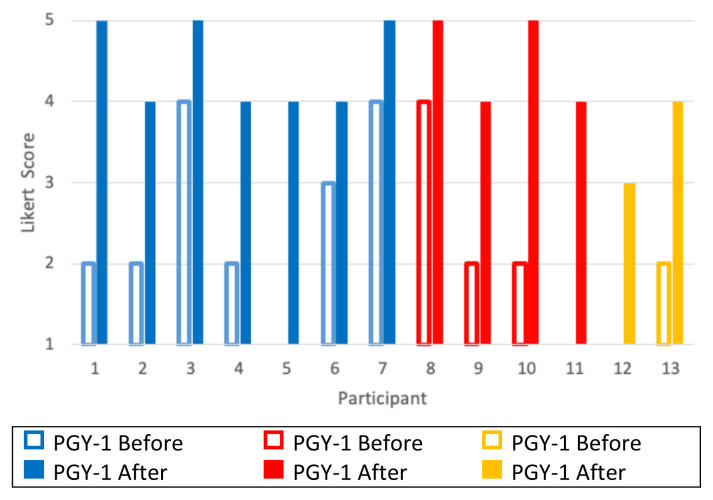
Learners describe familiarity with **MANAGEMENT** of bicarbonate toxicity before and after this oral boards case by Likert scale (1 = very unfamiliar, 5 = very familiar). Post graduate year, PGY.

Residents reported a score of 1 regarding familiarity of management of sodium bicarbonate toxicity on a Likert scale (1–5, 1 = very unfamiliar, 5 = very familiar) before the oral boards case and 4 and 5 after the oral boards case (Figure 3).

Other feedback from the learners included:

*Made me think about something I was not familiar with at all*

*Very great case and also very unique*

*Interesting and unexpected*

*The QTc was tough*

*Very broad differential and going through it taught me how to manage bicarbonate toxicity, of which I previously had no knowledge*

*I liked learning about a less known diagnosis*

*Realistic depiction of what patients in the ED will tell you*

Most of the residents regarded the case as difficult, but nonetheless, also considered it a welcome method of learning about sodium bicarbonate toxicity and systemic alkalosis. Based on feedback about the critical action of the prolonged QTc being difficult to diagnose when reading our original electrocardiogram, we introduced a different electrocardiogram with a more clearly prolonged QTc for distribution of this case, which would prompt a clinician towards clinical intervention and therefore a learner to appreciate the need to intervene in this oral boards case, considering it is a critical action. Novice learners may require increased utilization of the cues, but that does not take away from the impact of this modality of teaching diagnosis and management of sodium bicarbonate toxicity.

Because of the topic’s rare presentation, we feel that this oral boards case makes an excellent review of test-taking strategy and content review for residents to prepare for clinical practice and to prepare for taking the oral board exam. We have formatted the case for oral boards testing; however, with minor modifications, the case could potentially be used for medical simulation education or small group discussions. There is value in learning the approach to navigating the case; however, the information presented during the debriefing after the case is important to solidify the understanding of application of the learning points made throughout the case.

### Pearls

#### Sodium Bicarbonate Toxicity Signs and Symptoms

**Figure:**
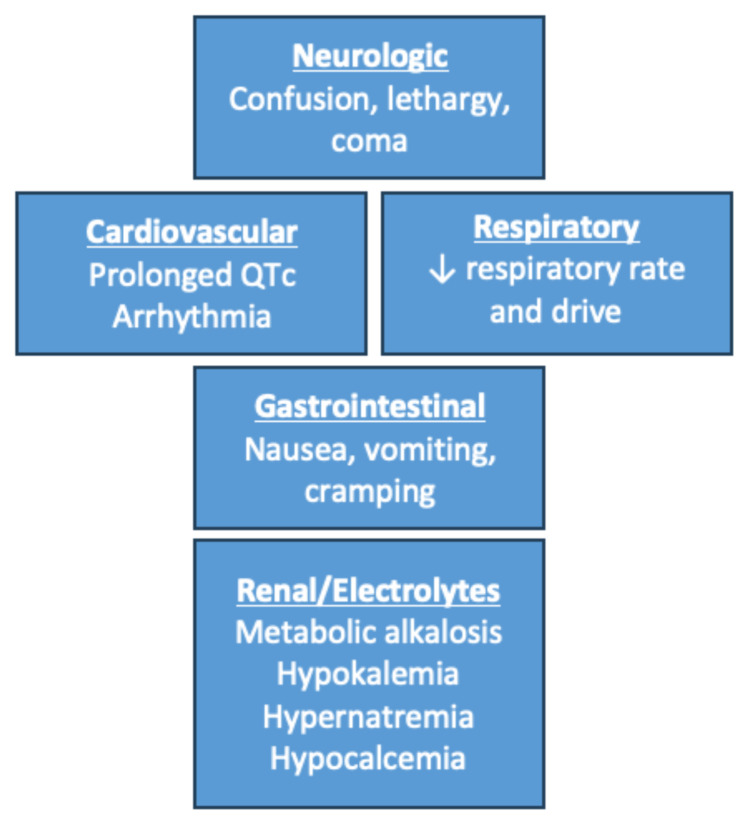
Image Source: Authors’ own image.

##### Management of Sodium Bicarbonate Toxicity

- Aggressive intravenous fluids
  ○ Hemodialysis if the patient cannot tolerate
- Address electrolyte abnormalities
- Airway intubation based on mental status or propensity to vomit
- Therapeutic agents
  ○ Acetazolamide

## Figures and Tables

**Table:** 

**Vitals:**	HR 72	BP 124/79	RR 10	Temp 97.2°F	O_2_Sat 96% (room air)

**Table:** 

	Milestone	Did not achieve level 1	Level 1	Level 2	Level 3
**1**	**Emergency Stabilization (PC1)**	□ **Did not achieve Level 1**	□ **Recognizes abnormal vital signs**	□ **Recognizes an unstable patient, requiring intervention** **Performs primary assessment** **Discerns data to formulate a diagnostic impression/plan**	□ **Manages and prioritizes critical actions in a critically ill patient** **Reassesses after implementing a stabilizing intervention**
**2**	**Performance of focused history and physical (PC2)**	□ **Did not achieve Level 1**	□ **Performs a reliable, comprehensive history and physical exam**	□ **Performs and communicates a focused history and physical exam based on chief complaint and urgent issues**	□ **Prioritizes essential components of history and physical exam given dynamic circumstances**
**3**	**Diagnostic studies (PC3)**	□ **Did not achieve Level 1**	□ **Determines the necessity of diagnostic studies**	□ **Orders appropriate diagnostic studies** **Performs appropriate bedside diagnostic studies/procedures**	□ **Prioritizes essential testing** **Interprets results of diagnostic studies** **Considers risks, benefits, contraindications, and alternatives to a diagnostic study or procedure**
**4**	**Diagnosis (PC4)**	□ **Did not achieve Level 1**	□ **Considers a list of potential diagnoses**	□ **Considers an appropriate list of potential diagnosis** **May or may not make correct diagnosis**	□ **Makes the appropriate diagnosis** **Considers other potential diagnoses, avoiding premature closure**
**5**	**Pharmacotherapy (PC5)**	□ **Did not achieve Level 1**	□ **Asks patient for drug allergies**	□ **Selects an appropriate medication for therapeutic intervention, considering potential adverse effects**	□ **Selects the most appropriate medication(s) and understands mechanism of action, effect, and potential side effects** **Considers and recognizes drug-drug interactions**
**6**	**Observation and reassessment (PC6)**	□ **Did not achieve Level 1**	□ **Reevaluates patient at least one time during the case**	□ **Reevaluates patient after most therapeutic interventions**	□ **Consistently evaluates the effectiveness of therapies at appropriate intervals**
**7**	**Disposition (PC7)**	□ **Did not achieve Level 1**	□ **Appropriately selects whether to admit or discharge the patient**	□ **Appropriately selects whether to admit or discharge** **Involves the expertise of some of the appropriate specialists**	□ **Educates the patient appropriately about their disposition** **Assigns patient to an appropriate level of care (ICU/Tele/Floor)** **Involves expertise of all appropriate specialists**
**22**	**Patient centered communication (ICS1)**	□ **Did not achieve level 1**	□ **Establishes rapport and demonstrates empathy to patient (and family)** **Listens effectively**	□ **Elicits patient’s reason for seeking health care**	□ **Manages patient expectations in a manner that minimizes potential for stress, conflict, and misunderstanding.**
**23**	**Team management (ICS2)**	□ **Did not achieve level 1**	□ **Recognizes other members of the patient care team during case (nurse, techs)**	□ **Communicates pertinent information to other healthcare colleagues**	□ **Communicates a clear, succinct, and appropriate handoff with specialists and other colleagues** **Communicates effectively with ancillary staff**

**Table:** 

**#1**	**Patient information form**
**#2**	**Electrocardiogram (ECG) from today’s hospital visit**
**#3**	**ECG from hospital visit one year prior**
**#4**	**Basic metabolic panel**
**#5**	**Complete blood count**
**#6**	**Urinalysis**
**#7**	**Venous blood gas**
**#8**	**Other lab studies**
**#9**	**Computed Tomography (CT) scan of head without contrast**
**#10**	**CT scan of abdomen and pelvis with contrast**
**#11**	**Cerebral Spinal Fluid Analysis**

**Table:** 

**Patient’s Name:**	**Mr. Genasis**
**Age:**	**37-years-old**
**Gender:**	**Male**
**Chief Complaint:**	**Nausea, vomiting, abdominal pain**
**Person Providing History: Emergency medical services (EMS), the patient’s partner, and the patient**	
**Vital Signs:**	
**Temperature:**	**97.2° Fahrenheit**
**Blood Pressure:**	**124/79 mmHg**
**Heart Rate:**	**72 beats per minute**
**Respiratory Rate:**	**10 breaths per minute**
**Pulse Oximetry:**	**96% on room air**
**Weight:**	**100 kilograms**

**Figure:**
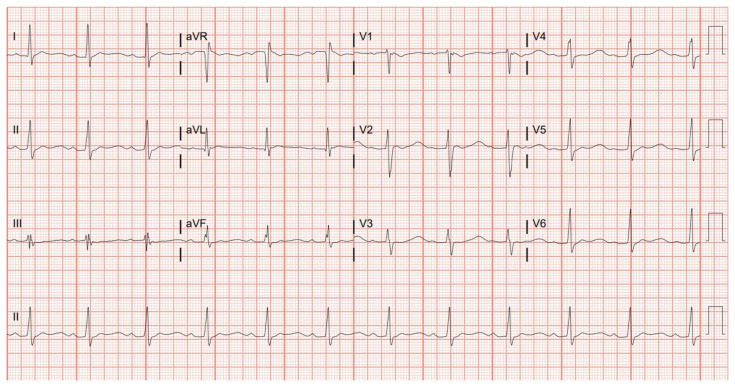
Image Source: Authors’ own image.

**Figure:**
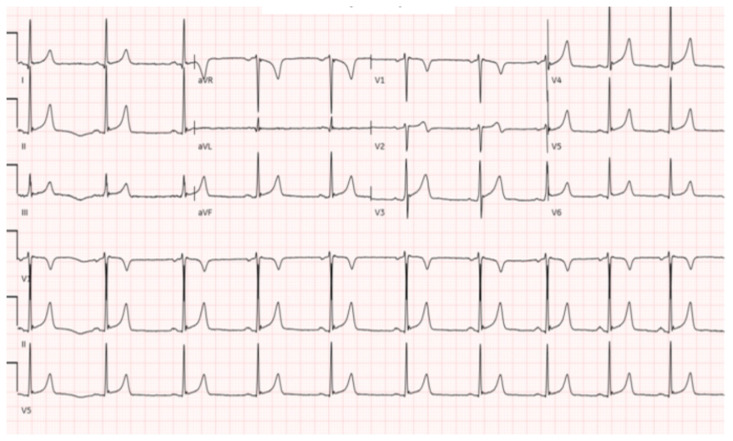
Image Source: Authors’ own image

**Table:** 

Na	153 mEq/L
K	2.9 mEq/L
Cl	110 mEq/L
CO2	40 mEq/L
BUN	10 mg/dL
Cr	1.2 mg/dL
Glucose	163 mg/dL
Phos	0.8 mg/dL
Mg	2.5 mg/dL
Ca	5.5 mg/dL

**Table:** 

WBC	9.0 × 10^3^/μL
Hgb	14 g/dL
Hct	50%
Platelets	300 × 10^3^/μL

**Table:** 

Appearance	Clear
Color	Yellow
Glucose	Negative
Ketones	Negative
Specific Gravity	1.02
Blood	Negative
pH	>9.0
Protein	30
Nitrite	Negative
Leukocyte	Trace
WBC	13/HPF
RBC	0/HPF
Squamous	Cells Occasional

**Table:** 

pH	7.52
pCO2	50 mmHg
pO2	140 mmHg
HCO3	41 mmol/L
Base Excess	+16
Lactate	3.3 mmol/L

**Table:** 

Liver Function Tests	Within Normal Limits
High Sensitivity Troponin	10 mg/L
COVID-19 Rapid Antigen Test	Negative

**Figure:**
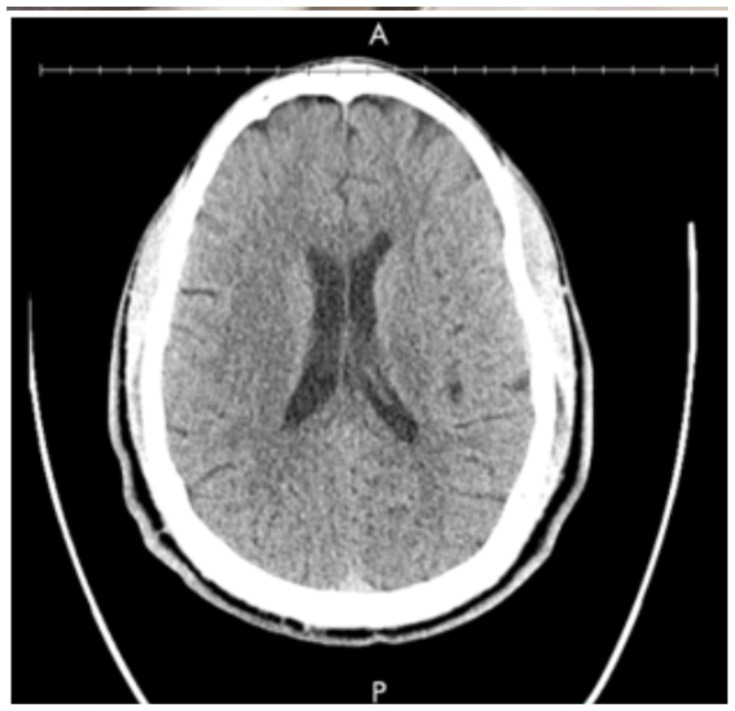
Image Source: Authors’ own image. No acute findings

**Figure:**
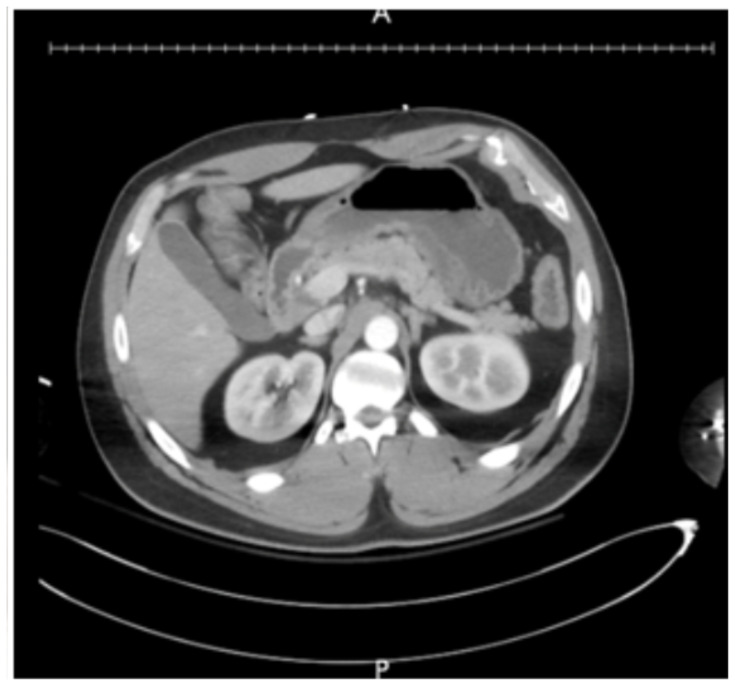
Image Source: Authors’ own image. No acute findings

**Table:** 

Appearance	Clear
Opening pressure	Normal
RBC Count	5 cells ×10^6^/L
WBC Count	3 cells ×10^6^/L
Glucose	Normal
Protein	0.3 g/L
Gram stain	No organisms

## FOR EXAMINER ONLY

### Oral Case Summary

**Diagnosis: Sodium Bicarbonate Intoxication**

**Case Summary:** A 37-year-old man presented to a community emergency department with nausea, vomiting, abdominal pain, and encephalopathy. Laboratory testing revealed hypernatremia, hypokalemia, hypocalcemia, and metabolic alkalosis. Electrocardiogram (ECG) was remarkable for a new prolonged QTc interval. The learners should discover that the patient reported drinking an unknown amount of baking soda prior to arrival typically after they have identified a metabolic alkalosis or if they inquire intently about substances used. The patient will subsequently be admitted to the intensive care unit and further resuscitated with fluids and electrolytes with close cardiovascular and neurological monitoring. The patient was using baking soda to whiten his teeth but also ingesting it in order to “trick” the urine drug screen at the parole office.

**Order of Case:** This is a case of a 37-year-old male with reported history of human immunodeficiency virus (HIV) who is presenting to the emergency department (ED) with chief complaints of nausea, vomiting, and abdominal pain as reported by emergency medical services (EMS). Upon arrival, EMS should be asked for their report, which will reveal that the patient was waiting at a parole office for an appointment with his parole officer and a routine drug screen. Approximately 30 minutes before presenting to the ED, while at the parole office, he began to have non-bloody, non-bilious emesis with complaints of nausea with diffuse abdominal pain. EMS were subsequently called and responded to the scene. At the time of their arrival, the patient’s Glasgow Coma Scale (GCS) was noted to be 11 (eyes open spontaneously, no vocal response, obeys commands). Patient was in no acute distress, vital signs were within normal limits, patient was euglycemic and was subsequently transported to the ED without any interventions.

After gathering the report, vital signs should be obtained. Vital signs will indicate that the patient is hemodynamically stable. The learner should then attempt to get more history from the patient and EMS. When asked about medications, the patient should occasionally mumble incoherent words. It is a critical action to further investigate the patient’s history, particularly the medication history, and identify any supplements the patient may be taking. If the learner does not investigate further, EMS will prompt the learner by stating, “We also have a contact number for the patient’s partner.” The learner should then “call” the patient's partner and gather a history. The partner will state the patient’s history and add that they “think he is taking something to whiten his teeth or pass drug tests, either way, it’s ‘all-natural’ baking soda.” After taking a full history, the learner will perform a thorough physical exam, revealing a confused patient with no obvious signs of trauma. The patient was, however, noted to have two episodes of significant somnolence and apnea that did resolve with stimulation and did not recur following resuscitation as outlined below.

An electrocardiogram (ECG) and appropriate laboratory studies should be ordered, including a basic metabolic panel (BMP) and a venous blood gas (VBG), all three of which are critical actions. Additionally, the learner may consider ordering a computed tomography (CT) scan of the patient’s head and abdomen/pelvis, although these are not critical actions. The ECG pertaining to this patient’s visit can be compared with a prior ECG. If the learner does not ask for an old ECG, a nurse will approach the examinee stating, “It looks like the patient has been seen in our emergency department before.” Regardless, the learner should diagnose a prolonged QTc on the most current ECG. If the examinee does not request the necessary labs, they will also be prompted when the nurse asks if there are any other laboratory studies that the examinee would like to order.

Expected initial interventions following the laboratory draw include symptomatic control with IV antiemetics, H2 agonists, and resuscitation with a 1-liter (L) bolus of IV normal saline (NS). The patient will report mild symptomatic relief and some improvement of mental status.

The patient’s initial venous blood gas reveals a severe metabolic alkalosis. A BMP revealed hypernatremia and hypokalemia. A liver function panel, lipase, and complete blood count were within normal limits. Urinalysis revealed a pH of greater than 9.0. Initial lactic acid was 3.3 mMol/L, which if re-checked improved to 1.8 mMol/L following fluid resuscitation and symptomatic management.

Following the patient’s first 1L NS bolus, he should be given an additional 1L bolus of isotonic fluid, maintenance intravenous fluids (IVF), and electrolytes should be repleted (potassium phosphate, potassium chloride). With resuscitation, the patient’s mental status should continue to improve.

Ultimately, the patient when more alert can clarify that he ingested an unspecified amount of baking soda earlier that day to whiten his teeth, and he also did ingest a “large amount” of baking soda to “clear” illegal drugs from his system before meeting with his parole officer in case he received a urine drug test. Given the patient’s severe electrolyte abnormalities and episodes of somnolence and apnea, he was admitted to the medical intensive care unit (MICU).

**Disposition:** Admission to a medical intensive care unit

**Critical Actions:**

1. Obtain a full history including medications and substances used
2. Correctly interpret a prolonged QTc
3. Correctly interpret a metabolic alkalosis
4. Aggressive intravenous fluid resuscitation and electrolyte replacement
5. Admission to an intensive care unit

### Historical Information

**Chief Complaint:** Nausea, vomiting, abdominal pain

**History of Present Illness:** Mr. Genasis is a 37-year-old man with an unknown past medical history who presented to the emergency department brought in by emergency medical services (EMS) with acute onset nausea, vomiting, and abdominal pain. Per EMS, they were called by an office worker at the patient’s parole office because they noticed he started acting strangely in the waiting room and began to have non-bloody, non-bilious emesis with complaints of nausea and diffuse abdominal pain. At the time of EMS’ arrival, the patient’s Glasgow Coma Scale (GCS) was noted to be 11 (eyes open spontaneously, no vocal response, obeys commands). The patient was in no acute distress, and vital signs were within normal limits. The patient was euglycemic and was subsequently transported to the ED without any interventions.

**Past Medical History:** Human Immunodeficiency Virus (HIV)

**Past Surgical History:** Denies

**Patient’s Medications and Supplements:**

- Last routinely took highly active antiretroviral therapy (HAART) over one year ago
- Uses household baking soda “to clean and whiten his teeth” multiple times a day

**Allergies:** No known drug allergies

**Social history:**

- Smoking: Denies
- Tobacco: Denies
- Drug use: History of polysubstance abuse
- Currently on parole

**Family history:** No pertinent family history

### Physical Exam Information

**Weight:** 100 kg

**General appearance:** Lethargic, obese

**Primary survey:**

- **Airway:** Intact and unobstructed
- **Breathing:** Equal breath sounds bilaterally
- **Circulation:** 2+ radial pulses

**Physical examination:**

- **General appearance:** Lethargic, obese, seems confused
- **HEENT:** Normocephalic, atraumatic
  ○ **Eyes:** Pupils 3 mm reactive bilaterally, extraocular movements are intact
- **Neck:** Within normal limits
- **Chest:** Clear to auscultation bilaterally, non-labored
- **Cardiovascular:** Regular rate and rhythm, no murmur
- **Respiratory:** Lungs clear to auscultation bilaterally, unlabored breathing 10 breaths/minute
- **Abdominal/GI:** Soft, non-distended, moderate diffuse tenderness to palpation without rebound or guarding
- **Genitourinary:** Deferred
- **Rectal:** Deferred
- **Extremities:** Moves all extremities spontaneously, no deformities
- **Back:** No tenderness to palpation, no step-offs
- **Neuro:** Lethargic, oriented to self and location, moving all extremities Glasgow Coma Scale: Eyes – 3, Verbal – 3, Motor – 5
- **Skin:** Warm, intact, moist, no rash
- **Lymph:** No cervical lymphadenopathy
- **Psych:** Generally non-communicative, uncooperative, occasionally belligerent

### Critical Actions and Cueing Guidelines

1. **Critical Action 1: Obtain a full history including medications** *and* **substances used**
  Additional questioning during history intake is required to identify recent ingestion of sodium bicarbonate.
  Cueing Guideline:
  *If the learner has not yet addressed this critical action, prompt the learner by having the EMS worker say, “We also found a baggie full of white powder in the patient’s pocket, looks like baking soda or sugar.”*
2. **Critical Action 2: Correctly interpret a prolonged QTc**
  The learner will need to interpret a prolonged corrected QT interval (QTc). Though this can be identified by a cardiac rhythm strip, most learners will calculate a QTc by analyzing an ECG.
  Cueing Guideline:
  *If the learner has not yet addressed this critical action, the nurse should state, “That rhythm looks funny to me.”*
3. **Critical Action 3: Correctly interpret a metabolic alkalosis**
  The learner will need to order a basic metabolic panel and venous blood gas (VBG) or arterial blood gas (ABG) to identify metabolic alkalosis.
  Cueing Guideline:
  *If the learner has not yet addressed this critical action, the nurse should comment, “That BMP looks very strange to me,” and/or, “Are there any other tests that you’d like to order right now?”*
4. **Critical Action 4: Aggressive intravenous fluid resuscitation and electrolyte replacement**
  The primary medical intervention for sodium bicarbonate ingestion is large volume isotonic fluid infusions and electrolyte (particularly potassium) supplementation.
  Cueing Guideline:
  *If the learner has not yet addressed this critical action, the nurse should ask the learner, “What should we do to fix this?”*
5. **Critical Action 5: Admission to an intensive care unit**
  Patients with an acute sodium bicarbonate ingestion will require close hemodynamic monitoring, frequent serum laboratory tests, close monitoring of urine output, and monitoring for mental status.
  Cueing Guideline:
  *If the learner suggests discharge to home or admission to a lower level of care to the hospital, the nurse should ask, “Is there anything that will need to be monitored?”*

## ORAL BOARDS ASSESSMENT

*A Recipe for Disaster – Sodium Bicarbonate Toxicity*

Learner: _________________________________________

**Critical Actions:**

□ Obtain a full history including medications and substances used
□ Correctly interpret a prolonged QTc
□ Correctly interpret a metabolic alkalosis
□ Aggressive intravenous fluid resuscitation and electrolyte replacement
□ Admission to an intensive care unit

**Summative and formative comments:**

**Milestone assessment:**

## DEBRIEFING AND EVALUATION PEARLS

### Sodium Bicarbonate Overdose

Sodium bicarbonate is very common in many households and in many products. It lends itself to countless everyday uses including cooking, cleaning, and even medical or health uses. A brief internet search will advertise the compound being an effective treatment for an assortment of processes ranging from indigestion, urinary tract infections, and even as a supplement to increase muscle mass for bodybuilders.^2^ Studies have even suggested that ingestion in a clinically controlled and standardized dose may slow the progression of chronic kidney disease.^3^ Additionally, the use of intravenous sodium bicarbonate in the hospital setting is conventional in circumstances such as cardiopulmonary resuscitations, refractory systemic acidosis, and to remedy toxic exposures. Sodium bicarbonate is a substance used by both emergency physicians and the public on a regular basis.

Given this prevalence, it is therefore remarkable that cases of documented sodium bicarbonate overdoses are comparatively sparse. For example, between the years 2000 to 2012, there were 192 total cases reported to the California Poison Control System (an average of just 16 cases per year).^4^ While this may be reflective of the relatively safe dosage in products, consideration also must be given that this disease process may be underdiagnosed. Obtaining a thorough history not just of medications prescribed but of other exposures is critical in diagnosing this toxicity. Laboratory testing is necessary to determine metabolic acidosis. A cursory medical history from a patient may not reveal that a patient is purposefully taking sodium bicarbonate because if they have altered mental status, the history may not be reliable. However, many patients who may have normal mental status may overlook their use of sodium bicarbonate, not realizing that it is important to make the diagnosis of sodium bicarbonate toxicity causing a metabolic alkalosis.^5^ In particular, sodium bicarbonate has been described in those with substance abuse disorder suffering from gastritis seeking cheap and easy relief^6^ but it has also been described in an infant who was given sodium bicarbonate to “help the baby burp.^7^”

Regardless of the patient’s background, once there is realization that a patient has metabolic alkalosis, direct questioning or chart review may be more useful than typical open interviewing in order to determine sodium bicarbonate exposure causing toxicity.

The toxic dose of sodium bicarbonate varies because kidney function and glomerular filtration rate (GFR) will determine the ability of kidneys to compensate for the metabolic alkalosis. It is estimated that healthy individuals can tolerate a total of 1700mEq sodium bicarbonate daily.^8^

For reference, one serving of baking soda is one-eighth a teaspoon, or 59mEq each of sodium and bicarbonate.^9^ In comparison, a manufactured sodium bicarbonate tablet may contain 7.7mEq of sodium and 7.7 mEq of bicarbonate.^10^ By comparison, one 50mL ampule of 8.4% medical sodium bicarbonate has 50mEq each of sodium and bicarbonate.^11^ The toxic dose of sodium bicarbonate will be lower in a patient with a lower GFR, regardless of cause.

The most common case reports of sodium bicarbonate toxicity are due to excessive ingestion of baking soda,^6^,^12^ though even less common iatrogenic injections/infusions can similarly cause sodium bicarbonate toxicity.^13^

#### Diagnosis

As evident by this patient’s laboratory testing, severe electrolyte disturbances are often present with sodium bicarbonate toxicity. The most common electrolyte disturbances include hypernatremia, hypokalemia, hypophosphatemia, hypocalcemia, and hypomagnesemia.^14^ In metabolic alkalosis associated with surreptitious behaviors, alkaline urine (pH ≥7.0) with high urine anion gap (>50 mEq/L) suggests surreptitious vomiting or significant alkali exposure.^15^ With exogenous sodium bicarbonate ingestion, functioning kidneys compensate by initiating bicarbonate diuresis in the body’s attempt to maintain homeostasis.^14^ This is accompanied by loss of sodium, potassium, and chloride. Water diuresis follows in suit and can worsen renal perfusion and GFR.^14^ Alkalosis from ingestion also causes shifting of potassium from extracellular to intracellular stores, further worsening hypokalemia, the most imminently life-threatening electrolyte disturbance of ingestion.^16^,^17^ Hypocalcemia results from alkalosis, which causes an increase in protein binding affinity and decreases free calcium.^18^

These metabolic disturbances can lead to a myriad of complications.

- Gastrointestinal (GI) upset is often the predominant presenting symptom (i.e., nausea, vomiting, diarrhea, pain).^6^ Gastric rupture has even been described as a complication in several cases.^19^
- ECG abnormalities such as QTc prolongation and resulting arrhythmia (eg, ventricular arrhythmias) are not uncommon.^14^
- Encephalopathy may be related to metabolic derangements. There has been a case report of hemorrhagic encephalopathy resultant of significant metabolic alkalosis.^8^
  ○ Encephalopathy with GI upset may place the patient at an increased risk of aspiration and respiratory collapse.
- Bradypnea or apnea may occur as a compensatory respiratory process for metabolic alkalosis^7^,^20^

#### Management

Aggressive intravenous fluids (IVF) with isotonic fluids will help remedy a hypovolemic state to help resolve acute renal injury which will facilitate renal excretion of the excessive intravascular bicarbonate.^21^, ^22^

- There is not a clear protocol for fluid resuscitation but 2–3L IVF bolus with maintenance fluid infusion at 1.5 times the typical maintenance rate may be appropriate in individuals without chronic renal or cardiac disease.^5^
- Patients who cannot tolerate large intravenous fluid volumes secondary to comorbidities may benefit from urgent/emergent hemodialysis.

Address electrolyte abnormalities

There is no studied protocol. Rather, common measures for electrolyte management and replacement have been described.

- **Hypokalemia** – most imminent life-threatening complication
  ○ Intravenous (IV) replacement is commonly performed , but oral replacement can be considered if the patient does not have altered mental status or refractory vomiting with a goal of about 5.0 mEq/L.
    ■ One study described administering 60 mEq of IV KCl at 125 mL/hr to treat a serum potassium of 2.1mmoL/L with a favorable response.^23^
  ○ Potassium chloride can treat both hypokalemia and hypochloremia. Avoid potassium acetate or potassium citrate because these could potentiate metabolic alkalosis.
  ○ Potassium levels need to be checked frequently because “rebound” hyperkalemia is a phenomenon which may occur even with appropriate resuscitation for up to three weeks post-ingestion^24^
- **Hypernatremia** occurs due to the sodium in *sodium* bicarbonate and vomiting/diarrhea.
  ○ Volume resuscitation will typically resolve hypernatremia
    ■ Oral (or feeding tube) rehydration can be considered in a patient who does not have altered mental status. In patients with severe dehydration or shock, the initial step is fluid resuscitation with isotonic fluids.^25^
    ■ Correction of hypernatremia too rapidly can potentially lead to cerebral edema as a complication. The goal is to decrease serum sodium by not more than 12 mEq in 24 hours.^25^
- **Hypocalcemia** may occur in the alkalotic state reducing available ionized calcium due to increased binding of calcium to the albumin.^18^, ^26^
  ○ In the case of ECG changes (particularly a prolonged QTc), IV magnesium should be considered.^27^
  ○ IV calcium is recommended in cases of severe symptoms or a prolonged QTc.
  ○ Calcium gluconate 1–2g is often preferred to calcium carbonate’s risk for tissue extravasation.^27^ In addition, calcium carbonate would introduce more bicarbonate to the system.

Airway intubation should be considered.

- Particularly if the patient has refractory vomiting and/or is severely encephalopathic or has significant bradypnea or apnea.

Other therapeutic agents:

- Antiemetics may be used to potentially assist with refractory nausea/vomiting. Understandably, many antiemetics have been associated with causing a prolonged QTc with high doses, but one case documented successfully administering IV metoclopramide (10 mg) and ondansetron (8 mg) with favorable response and no complications noted.^23^
- Acetazolamide may lower the serum bicarbonate in severe toxicity by inhibiting carbonic anhydrase.
  ○ One may consider IV 500 mg acetazolamide administration for metabolic alkalosis refractory to IVF.^28^

Monitoring urine pH can be helpful to monitor effectiveness of treatment.

- Urine pH generally is <6.0 in patients with metabolic alkalosis because proximal reabsorption of bicarbonate and net acid excretion are increased to sustain the process.^29^
- Neutral urine pH is typically 6–7.
- However, in the recovery phase of volume contraction metabolic alkalosis, urine pH may become alkaline when correction of volume depletion with saline solution administration causes bicarbonate diuresis^15^

#### Disposition

The symptomatic patient will typically need inpatient monitoring on telemetry and care interventions dependent on the severity of electrolyte derangements, risk of decompensation, and the need for serial laboratory testing and ECGs, particularly if the patient is also having depressed breathing, depressed mental status, and ECG changes.^23^

It is also important to elucidate why the patient ingested sodium bicarbonate if that was the means of toxicity. If self-harm was the goal, precautions and psychiatric evaluation may be necessary.

Consider consults:

- Toxicology
- Nephrology
